# Risk factors of video urodynamics and bladder management for long-term complications in patients with chronic spinal cord injury

**DOI:** 10.1038/s41598-024-63441-w

**Published:** 2024-06-02

**Authors:** Yu-Chen Chen, Hann-Chorng Kuo

**Affiliations:** 1 Graduate Institute of Clinical Medicine, College of Medicine, Kaohsiung Medical University, Kaohsiung, Taiwan; 2 Department of Urology, Kaohsiung Medical University Hospital, Kaohsiung Medical University, Kaohsiung, Taiwan; 3 Regenerative Medicine and Cell Therapy Research Center, Kaohsiung Medical University, Kaohsiung, Taiwan; 4 Department of Urology, Hualien Tzu Chi Hospital, Buddhist Tzu Chi Medical Foundation, Tzu Chi University, No.707 Sec.3, Zhongyang Rd., Hualien City, 970473 Taiwan (R.O.C.)

**Keywords:** Spinal cord injuries, Urodynamics, Urinary catheterization, Neurogenic bladder, Complications, Self-catheterization, Risk factors, Urology

## Abstract

This study explores 15-year urological complications in chronic spinal cord injury (SCI) patients and investigates the predictive factors from video-urodynamic study (VUDS) and bladder management. Analyzing 864 SCI patients with a mean 15.6-year follow-up, we assessed complications and utilized multivariate logistic regression for risk evaluation. VUDS factors such as autonomic dysreflexia, detrusor sphincter dyssynergia, vesicourethral reflux (VUR), contracted bladder, and high voiding detrusor pressure significantly increased the likelihood of recurrent urinary tract infections (rUTI). Low bladder compliance, VUR, and contracted bladder notably raised the risk of hydronephrosis, while contracted bladder and detrusor overactivity with detrusor underactivity heightened chronic kidney disease risk. Volitional voiding reduced rUTI and VUR risk, whereas Valsalva maneuver-assisted voiding increased hydronephrosis risk. In conclusion, a contracted bladder identified in VUDS is associated with long-term urological complications in SCI, we propose that patients already experiencing a contracted bladder should prioritize volitional voiding as their preferred bladder management strategy to minimize the risk of additional complications such as rUTI and VUR. These findings unveil previously unexplored aspects in research, emphasizing the need for proactive management strategies in this patient population.

## Introduction

Patients with chronic spinal cord injury (SCI) often suffer from urological complications arising from neurogenic lower urinary tract dysfunction (NLUTD), disrupts normal bladder storage and voiding functions^1,2^. These challenges significantly impact their quality of life and overall health. Long-term urological complications in SCI patients encompass recurrent urinary tract infections (rUTI), hydronephrosis, vesicoureteric reflux (VUR), chronic kidney disease (CKD), bladder stones, renal stones, bladder cancers and urethral abscess among other issues.

Limited studies have examined the link between SCI and prolonged urological complications, underscoring the necessity for identification of risk factors, effective predictive measures and management strategies to alleviate these issues, especially focusing on complications lasting over ten years, because conducting a follow-up for such an extended duration in patients with SCI can be challenging^1,3^.

In response to identification of risk factors to urological complications, video urodynamic studies (VUDS) have emerged as valuable tools to assess voiding detrusor pressure, bladder compliance, sphincter function, bladder capacity, and other parameters, offering comprehensive insights into post-SCI bladder dynamics, which possibly guides management decisions^4,5^. Furthermore, bladder management practices, including volitional voiding and Valsalva maneuvers, have demonstrated notable impacts on the incidence of certain urological complications^6^.

Despite advancements in understanding, comprehensive research analyzing the long-term implications of VUDS parameters and bladder management on urological complications in SCI patients remains limited. Understanding these risk factors identified through VUDS and the impact of diverse bladder management strategies is crucial for devising tailored interventions and preventive measures to mitigate long-term urological complications in individuals with chronic SCI. Therefore, this study aims to investigate the relationship between VUDS findings, bladder management techniques, and the occurrence of urological complications during extended follow-up periods in SCI patients in the large cohort.

## Patients and methods

From September 1997 to February 2022, patients with SCI who underwent their initial VUDS during the first medical consultation post-injury at a single tertiary hospital in Taiwan, and received regular follow-ups, were included. The initial assessment encompassed neuro-urological evaluations including physical examinations, renal and bladder sonography, urinalysis, and VUDS. The SCI level was classified as cervical, thoracic, lumbar, or sacral based on history and neurological examinations^7^. Baseline demographic data and the level of SCI impairment were recorded.

Primary outcomes comprised VUDS parameters^8^, such as the occurrence of autonomic dysreflexia (AD) during VUDS, detrusor sphincter dyssynergia (DSD), low bladder compliance (defined as < 10 ml/cmH_2_O), presence of VUR, contracted bladder (defined as having small bladder capacity with a deformed bladder shape and low bladder compliance), high detrusor pressure during voiding phase (defined as Pdet.Qmax > 40 cmH_2_O in male SCI and Pdet.Qmax > 30 cmH_2_O in female SCI patients), detrusor overactivity (DO), detrusor overactivity with detrusor underactivity (DO-DU), detrusor underactivity (DU), detrusor acontractile (DA), and various bladder management techniques including volitional voiding, using a diaper, voiding with Valsalva maneuver, voiding with percussion, reflex voiding, clean intermittent catheterization (CIC), urethral catheter, or cystostomy^6^. All methods, definitions, and units adhered to the standards recommended by the ICS^9^. Secondary outcomes included long-term urological complications such as recurrent urinary tract infections (rUTI), hydronephrosis (detected via ultrasound), chronic kidney disease (CKD), VUR, renal stones, bladder stones, bladder cancer, and urethral abscess. The definition of UTI was aligned with current clinical practice according to the 2024 EAU Neuro-urology guidelines^10^, which include the following criteria: UTI is defined as the onset of signs and/or symptoms accompanied by laboratory findings of a UTI (bacteriuria, leukocyturia, and positive urine culture). Significant bacteriuria is considered present in individuals performing intermittent catheterization (IC) with > 10^2^ cfu/mL, > 10^4^ cfu/mL in clean-void specimens, and any detectable concentration in suprapubic aspirates. The signs and symptoms of a UTI in those with neuro-urological disorders include fever, new onset or increase in incontinence (including leaking around an indwelling catheter), increased spasticity, malaise, lethargy or sense of unease, cloudy urine with increased odor, discomfort or pain over the kidney or bladder, dysuria, or autonomic dysreflexia (AD). rUTI referred to ≥ 2 infections in six months or ≥ 3 infections in 1 year^11^. Hydronephrosis was defined by renal sonography revealing dilatation of the renal pelvis and calyces. CKD was defined as the presence of kidney damage or an estimated glomerular filtration rate (eGFR) persisting below 60 ml/min per 1.73 square meters for 3 months or more^12^.

Patients with incomplete medical records were excluded. Data were approximately normally distributed and presented as means with standard deviations (SD). Differences among groups were analyzed using the chi-square test for categorical variables or analysis of variance test for continuous variables. All tests were two-sided, with p values ≤ 0.05 considered statistically significant. Univariate and multivariate logistic regression analyses using forward selection were conducted to assess the association between VUDS-related parameters, bladder management, and the occurrence of complications during long-term follow-up. Statistical analyses were performed using "IBM SPSS Statistics," version 26. This study received approval from the Buddhist Tzu Chi General Hospital Research Ethics Committee (IRB110-033-B). The requirement for informed consent was waived by the Buddhist Tzu Chi General Hospital Research Ethics Committee (IRB110-033-B) due to its nature as a minimal-risk retrospective chart review, where there will be no patient interaction. All research was performed in accordance with relevant guidelines and regulations.

### Ethical approval

This study was approved by the Research Ethics Committee of the Buddhist Tzu Chi General Hospital (IRB110-033-B).

## Results

Reviewing the records VUDS, we identified 16 patients who underwent examination but did not continue with urological management or follow-up at our clinic. Among them, there were 10 men and 6 women, ranging in age from 25 to 67 years old. This group included 2 patients with cervical SCI, 5 with thoracic SCI (below T6), 7 with lumbar SCI, and 2 with lesions below the sacrum. Most of these patients had undergone VUDS examinations before the year 2000, resided outside the city where our hospital is located, and could not be contacted for updates on their status and urological complications. Consequently, their data were excluded from the final analysis.

Out of the final 864 enrolled patients, 654 (75.7%) were male, and 210 (24.3%) were female with SCI. The mean follow-up duration was 15.6 ± 9.9 years. 37.8% of SCI patients suffered from cervical lesions. The occurrence of complications varied across different SCI injury levels as shown in Table 1. AD and bladder stones significantly differed among the five groups. Specifically, AD was significantly higher in cervical lesions compared to the thoracic > 6 group and thoracic < 6 group, while bladder stones were significantly more prevalent in patients with thoracic > 6 than those below the sacrum.

**Table 1:** The distribution of long-term complications among different SCI levels.

Complications	Cervical (N = 327)	Thoracic < 6 (N = 149)	Thoracic > 6 (N = 227)	Lumbar^1^ (N = 132)	Sacral and below sacral^2^ (N = 29)	*p*-value
Recurrent UTI	249 (76.1)	111 (74.5)	156 (68.7)	92 (69.7)	16 (55.2)	0.064
AD	178 (55.4)^a,b^	21 (14.1)^a^	4 (1.8)^b^	0	0	< 0.001*
Hydronephrosis	34 (10.4)	27 (18.1)	32 (14.1)	17 (12.9)	6 (20.7)	0.144
CKD	7 (2.1)	5 (3.4)	11 (4.8)	2 (1.5)	0	0.311
VUR	19 (5.8)	7 (4.7)	8 (3.5)	10 (7.6)	4 (13.8)	0.146
Bladder cancer	4 (1.2)	4 (2.7)	0	0	0	0.077
Bladder stone	12 (3.7)	15 (10.1)^c^	9 (4.0)	8 (6.1)	1 (3.4)^c^	0.044*
Renal stone	13 (4.0)	6 (4.0)	11 (4.8)	3 (2.3)	1 (3.4)	0.829
Urethral abscess	2 (0.6)	2 (1.3)	1 (0.4)	1 (0.8)	0	0.834

Data are presented as number (% of patients) of patients with specific urological complication in patients with chronic SCI of different level.

*Significant difference (p < 0.05) between different SCI levels.

^a^Significant difference between cervical level and thoracic > 6 level.

^b^Significant difference between cervical level and thoracic < 6 level.

^c^Significant difference between thoracic > 6 level and below sacrum level.

^1^The spinal cord injury is located between L1 to L5.

^2^Damage includes both the sacral level and areas below the sacral level.

*SCI* spinal cord injury, *UTI* recurrent urinary tract infection, *AD* autonomic dysreflexia, *CKD* chronic kidney disease, *VUR* vesicourethral reflux.

**Figure 1:**
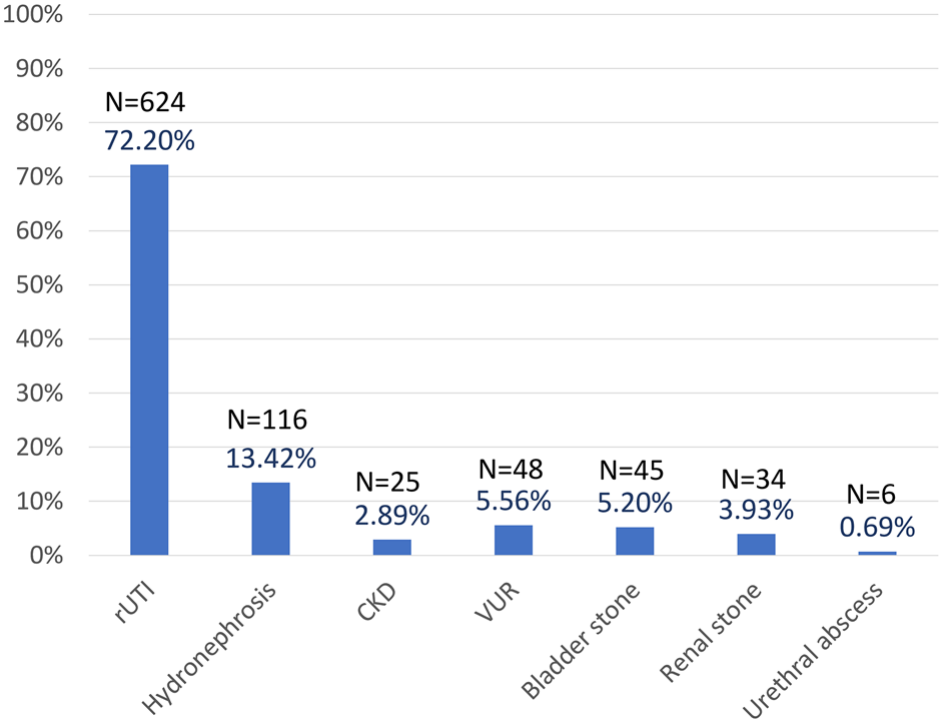
The percentage and the number of different long-term urological complications in our cohort (N = 864 patients). *rUTI* recurrent urinary tract infection, *CKD* chronic kidney disease, *VUR* vesicourethral reflux.

The most prevalent long-term urological complication was rUTI, experienced by 72.2% of patients, while CKD/ESRD affected only 2.89% of patients (Fig. 1). Table 2 outlines VUDS findings and bladder management associated with each complication, identifying specific risk factors for VUDS parameters and bladder management techniques. Supplementary Table 1 demonstrated the risk factors, including VUDS parameters and bladder management for the long-term complications. Multivariate logistic regression analysis further revealed that AD, DSD, VUR, contracted bladder, and high voiding detrusor pressure at Qmax (Pdet.Qmax) during initial VUDS significantly increased the risk of rUTI (adjusted odds ratio: 1.780, 1.535, 2.028, 3.156, 1.482, respectively; confidence intervals: 1.172–2.702, 1.041–2.263, 1.018–4.041, 2.017–4.940, 1.008–2.179, respectively), while volitional voiding significantly reduced the risk of rUTI (adjusted odds ratio: 0.407). Low bladder compliance, VUR, and contracted bladder and voiding with Valsalva maneuvers significantly raised the risk of hydronephrosis (adjusted odds ratio: 2.065, 10.342, 4.00, 2.033 respectively; confidence intervals: 1.098–3.883, 6.092–17.555, 2.470–6.477, 1.180–3.502, respectively). Contracted bladder and DO-DU significantly increased the risk of CKD (adjusted odds ratio: 4.975, 9.148, respectively; confidence intervals: 2.110–1.731, 1.724–48.554, respectively). The presence of VUR at initial VUDS and voiding on diaper significantly heightened the risk of long-term VUR (adjusted odds ratio: 1364, 3.458, respectively; confidence intervals: 173–10,716, 1.274–9.383, respectively), volitional voiding significantly reduced the risk of VUR (adjusted odds ratio 0.153; confidence intervals: 0.045–0.525).

**Table 2:** The number and percentage of initial VUDS findings and bladder managements in different long-term urological complications in patients with chronic spinal cord injury.

Factors	rUTI (n = 624)	Hydronephrosis (n = 116)	CKD (n = 25)	VUR (n = 48)	Bladder stone (n = 45)	Renal stone (n = 34)
VUDS findings						
AD	219 (35.1)*	39 (33.6)	5 (20.0)	19 (39.6)	14 (31.1)	14 (41.2)
DSD	466 (74.7)*	82 (70.7)	20 (80.0)	38 (79.2)	31 (68.9)	24 (70.6)
Low compliance	64 (10.3)*	26 (22.4)*	5 (20.0)	6 (12.5)	9 (20.0)*	7 (20.6)*
Presence of VUR	77 (12.3)*	50 (43.1)*	2 (8.0)	47 (97.9)*	9 (20.0)*	8 (23.5)*
Contracted bladder	219 (35.1)*	75 (64.7)*	16 (64.0)*	30 (62.5)*	22 (48.9)*	20 (58.8)*
High voiding Pdet.Qmax	289 (46.5)*	55 (47.4)	13 (52.0)	22 (45.8)	17 (37.8)	13 (38.2)
Detrusor contractility						
DO	445 (71.3)	81 (69.8)	18 (72.0)*	38 (79.2)	25 (55.6)	23 (67.6)
DO-DU	8 (1.3)	1 (0.9)	2 (8.0)*	0	1 (2.2)	1 (2.9)
DU/DA	171 (27.4)	34 (29.3)	5 (20.0)	10 (20.8)	19 (42.2)	10 (29.4)
Bladder managements						
Volitional voiding	88 (14.1)*	15 (12.9)	7 (28.0)	6 (12.5)	4 (8.9)	6 (17.6)
On diaper	304 (48.7)	64 (55.2)	15 (60.0)	27 (56.3)	19 (42.2)	20 (58.8)
Abdominal pressure	112 (17.9)	27 (23.3)	5 (20.0)	8 (16.7)	10 (22.2)	7 (20.6)
Percussion	193 (30.9)	32 (27.6)	10 (40.0)	12 (25.0)	8 (17.8)	7 (20.6)
Reflex voiding	116 (18.6)	24 (20.7)	8 (32.0)*	11 (22.9)	5 (11.1)	4 (11.8)
CIC	112 (17.9)	24 (20.7)	3 (12.0)	14 (29.2)*	3 (6.7)	1 (2.9)
Urethral catheter	98 (15.7)	18 (15.5)	3 (12.0)	8 (16.7)	11 (24.4)	6 (17.6)
Cystostomy	96 (15.4)*	15 (12.9)	3 (12.0)	8 (16.7)	13 (28.9)*	6 (17.6)

Data are presented as number (% of patients) of patients with specific VUDS findings or bladder managements among complications.

*Significant difference (p < 0.05) in the percentage of the VUDS finding or bladder management between with and without urological complications.

*VUDS* video urodynamic study, *AD* autonomic dysreflexia, *DSD* detrusor sphincter dyssynergia, *VUR* vesicourethral reflux, *Pdet.Qmax* detrusor pressure at Qmax, *DO* detrusor overactivity, *DO-DU* detrusor overactivity with detrusor underactivity, *DU/DA* detrusor underactivity/acontractile detrusor, *CIC* clean intermittent catheterization, *CKD* chronic kidney disease.

## Discussion

While prior literature extensively addresses urological complications in SCI patients^13^, few studies delve deeply into the correlations among VUDS parameters^1,14,15^, bladder management strategies^6,16^, and long-term complications. Existing research often focuses on isolated aspects of post-SCI urological issues, lacking comprehensive evaluations of VUDS parameters, associated bladder management, and their influence on diverse long-term complications.

Our study presented novel insights by (1) delineating the distribution of long-term urological complications across different SCI levels in a large cohort of 864 patients with SCI in Taiwan, (2) establishing an association between initial VUDS findings and various long-term urological complications over a mean follow-up of 15.6 years, and (3) identifying VUDS-related and bladder management-related risk factors that predict long-term urological complications through multivariate regression. This analysis aimed to provide clinical guidance to prevent long-term complications by minimizing confounding factors.

NLUTD gradually leads to urological complications^17–19^, and the level of SCI is closely tied to these complications^20,21^. AD is a well-known to occur in association with SCI above T6, consistent with our findings. However, our study revealed 1.8% of patients with SCI below T6 experiencing AD, emphasizing the continued risk of AD in such patients, especially during urodynamic surveys, which is consistent with the concern in the study by Canon et al.^22^.

In our study, 72.2% of SCI patients experienced at least one episode of rUTI, a common finding consistent with a 40-year follow-up study of 43 SCI patients reporting 100% suffering from rUTI^3^. While clinical parameters and bladder management without urodynamic-related parameters were utilized in risk prediction models for UTI in the recent study^23^, VUDS remains crucial for evaluating bladder function, sphincter function, and Pdet in SCI patients^7,14,24^. Our study indicated that rUTI might stem from unfavorable bladder conditions, such as elevated Pdet, large residual urine, VUR, contracted bladder, or other lower urinary tract abnormalities identified during initial VUDS post SCI. Furthermore, our findings suggested that utilizing volitional voiding as a bladder management strategy reduced the risk of rUTI, which is consistent with previous studies^6,25^.

Concerning upper urinary tract damage, urodynamic study-related risk factors such as detrusor overactivity (DO) with DSD, Pdet, poor bladder compliance, and VUR have been previously established^14,15^. A systematic review has indicated that poor bladder compliance and high Pdet are major contributors to hydronephrosis^14^, aligning with the findings of our present study. However, our research identified additional risk factors, specifically contracted bladder, associated with both hydronephrosis and CKD. While evidence has indicated that voiding with a Valsalva pattern should be avoided in SCI patients with a leak-point pressure exceeding 40 cmH_2_O to mitigate urological complications^6^, our study further emphasizes this point. Specifically, we found that voiding with a Valsalva pattern poses a risk for hydronephrosis, even after adjusting for various factors in multivariate analysis, including video-urodynamic study parameters and bladder management strategies.

Our study, with a mean follow-up of 15.6 years and 864 SCI patients, contributes valuable insights. In comparison, other studies with smaller cohorts and varying follow-up durations indicated different risk factors for urological complications^1,3,14^. Our research focused on the interrelationship between initial VUDS findings, bladder management, and long-term complications in SCI patients, offering critical clinical guidance for different bladder management approaches in this population, especially with poor initial VUDS findings. Specifically, our findings emphasized that even with unfavorable VUDS-related factors, such as AD, DSD, VUR, contracted bladder, and high Pdet, utilizing volitional voiding if feasible can reduce the risk of rUTI. Moreover, avoiding voiding with abdominal pressure, especially in patients with poor VUDS parameters, can help prevent hydronephrosis. Lastly, our study highlighted that volitional voiding, if feasible, serves as a protective factor against the occurrence of VUR, whereas voiding with a condom catheter posed a risk.

In summary, unlike prior studies focusing on specific VUDS parameters, our research comprehensively assessed a wide range of VUDS-related parameters and bladder management strategies collectively. This thorough evaluation provided a more nuanced understanding of how these factors collectively contribute to diverse long-term complications. Collectively, these findings emphasize the need for tailored management strategies to enhance patient outcomes. However, the retrospective nature of our study, the absence of data of hospitalization due to febrile UTI, and the absence of acute measurements for parameters such as detrusor leak point pressure, amplitude of detrusor overactivity (DO), and maximum detrusor pressure during the storage phase, pose limitations, underscoring the necessity for further prospective research to validate these associations and provide definitive evidence for personalized management approaches in this patient population. This study reveals the urological complication can occur in patients with different VUDS findings and different bladder management. Therefore, the urological complications should be monitoring in patients with chronic SCI in long-term follow-up.

The limitation of the study is not all urodynamic characteristic parameters were collected and used for analysis of the risks for the urological complications in patients with chronic SCI such as detrusor leak point pressure, amplitude of DO during storage phase, and maximum detrusor pressure using storage phase. In addition, we do not have VUDS data for all 864 patients during the last examination because not every patient was suitable for or willing to undergo VUDS. Another limitation is that we cannot provide full patient characteristics for analysis of VUDS findings and urological complications because the patients data were collected across a 25 years span, therefore, some early clinical data have been disappeared. Lastly, the observational nature of our study emphasizes the need for further prospective research to validate our findings.

## Conclusions

Our study conducted an extensive analysis of VUDS parameters and various bladder management strategies, elucidating their associations with a spectrum of urological complications in a large cohort with a mean follow-up of 15.6 years. A contracted bladder was identified as a significant VUDS-related risk factor for long-term urological complications, including rUTI, hydronephrosis, and CKD. Volitional voiding, if feasible, serves as a protective factor against the occurrence of long-term rUTI and VUR. These findings shed light on three distinct aspects not extensively explored in prior studies.

### Supplementary Information

Supplementary Table 1.

## Data Availability

The datasets used and/or analysed during the current study available from the corresponding author on reasonable request.
