# Supplementary material for: Risk factors of video urodynamics and bladder management for long-term complications in patients with chronic spinal cord injury
Source: Sci Rep. 2024 Jun 2;14:12632. doi: 10.1038/s41598-024-63441-w (PMC11144236; doi:10.1038/s41598-024-63441-w)
Supplement: Supplementary file 1 — Supplementary Table 1. [file 41598_2024_63441_MOESM1_ESM.docx]

**Supplementary table 1. Univariable and multivariable logistic regression analyses of predictors for VUDS-related parameters and bladder managements associated with different long-term complications**

|  | **rUTI** | | | | **Hydronephrosis** | | | | **CKD** | | | | **VUR** | | | |
| --- | --- | --- | --- | --- | --- | --- | --- | --- | --- | --- | --- | --- | --- | --- | --- | --- |
| **Predictive factors** | **Univariate** | | **Multivariate** | | **Univariate** | | **Multivariate** | | **Univariate** | | **Multivariate** | | **Univariate** | | **Multivariate** | |
|  | Odds ratio (95% CI) | *p*-value | Odds ratio (95% CI) | *p*-value | Odds ratio (95% CI) | *p*-value | Odds ratio (95% CI) | *p*-value | Odds ratio (95% CI) | *p*-value | Odds ratio (95% CI) | *p*-value | Odds ratio (95% CI) | *p*-value | Odds ratio (95% CI) | *p*-value |
| **VUDS variables** |  |  |  |  |  |  |  |  |  |  |  |  |  |  |  |  |
| AD | 2.704  (1.854-3.942) | <0.001 | 1.780  (1.172-2.702) | 0.007 | 1.216  (0.802-1.843) | 0.358 | - | - | 0.576  (0.214-1.551) | 0.275 |  |  | 1.572 (0.865-2.859) | 0.138 | - | - |
| DSD | 2.333  (1.707-3.188) | <0.001 | 1.535  (1.041-2.263) | 0.031 | 1.071  (0.697-1.644) | 0.754 | - | - | 1.786  (0.663-4.811) | 0.251 |  |  | 1.717 (0.843 -3.501) | 0.137 | - | - |
| Low bladder compliance | 2.629  (1.326-5.209) | 0.006 | - | - | 4.213  (2.491-7.124) | <0.001 | 2.065 (1.098-3.883) | 0.024 | 2.790  (1.016-7.663) | 0.047 |  |  | 1.571 (0.645-3.829) | 0.32 | - | - |
| Presence of VUR | 2.931  (1.529-5.615) | 0.001 | 2.028  (1.018-4.041) | 0.045 | 14.155  (8.659-23.139) | <0.001 | 10.342 (6.092-17.555) | <0.001 | 0.761  (0.176-3.285) | 0.715 |  |  | 888.415 (119.580-6600.441) | <0.001 | 1364.119 (173.647-10716.082) | <0.001 |
| Contracted bladder | 4.094  (2.671-6.275) | <0.001 | 3.156  (2.017-4.940) | <0.001 | 6.126  (4.037-9.296) | <0.001 | 4.000 (2.470-6.477) | <0.001 | 4.679  (2.039-10.737) | <0.001 | 4.975 (2.110-1.731) | <0.001 | 4.601 (2.513-8.422) | <0.001 | - | - |
| High voiding Pdet | 2.108  (1.531-2.902) | <0.001 | 1.482  (1.008-2.179) | 0.045 | 1.311  (0.885-1.941) | 0.177 | - | - | 1.537  (0.693-3.410) | 0.29 |  |  | 1.198 (0.667-2.149) | 0.545 | - | - |
| Detrusor contractility |  |  |  |  |  |  |  |  |  |  |  |  |  |  |  |  |
| DO | - | 0.089 |  |  | - | 0.834 |  |  | - | 0.046 | - | 0.026 |  |  |  | 0.388 |
| DO-DU | 0.550  (0.177-1.707) |  |  |  | 0.532  (0.068-4.146) |  |  |  | 5.859  (1.209-28.386) |  | 9.148 (1.724-48.554) |  |  |  | 0.000 (0-0) |  |
| DU/DA | 0.717  (0.520-0.988) |  |  |  | 0.991  (0.644-1.524) |  |  |  | 0.650  (0.239-1.769) |  | 0.843 (0.304-2.336) |  |  |  | 0.606 (0.297-1.237) |  |
|  |  |  |  |  |  |  |  |  |  |  |  |  |  |  |  |  |
| **Bladder managements** |  |  |  |  |  |  |  |  |  |  |  |  |  |  |  |  |
| Spontaneous voiding | 0.482  (0.334-0.696) | <0.001 | 0.407  (0.272-0.608) | <0.001 | 0.681  (0.383-1.208) | 0.188 |  |  | 1.909  (0.783-4.655) | 0.155 |  |  | 0.672 (0.280-1.612) | 0.373 | 0.153 (0.045-0.525) | 0.003 |
| On diaper | 1.242  (0.921-1.676) | 0.156 | - | - | 1.445  (0.976-2.142) | 0.066 |  |  | 1.702  (0.756-3.833) | 0.199 |  |  | 1.468 (0.816-2.639) | 0.2 | 3.458 (1.274-9.383) | 0.015 |
| Abdominal pressure | 0.772  (0.535-1.114) | 0.167 | - | - | 1.341  (0.839-2.143) | 0.22 | 2.033 (1.180-3.502) | 0.011 | 1.061  (0.392-2.870) | 0.907 |  |  | 0.839 (0.385-1.829) | 0.66 | - | - |
| Percussion | 1.286  (0.919-1.798) | 0.142 | - | - | 0.897  (0.580-1.387) | 0.625 | - | - | 1.616  (0.716-3.647) | 0.248 |  |  | 0.786 (0.402-1.537) | 0.481 | - | - |
| Reflex voiding | 1.964  (1.239-3.113) | 0.004 | - | - | 1.407  (0.861-2.298) | 0.173 | - | - | 2.498  (1.057-5.906) | 0.037 |  |  | 1.569 (0.780-3.155) | 0.207 | - | - |
| CIC | 1.281  (0.848-1.936) | 0.239 | - | - | 1.326  (0.813-2.162) | 0.259 | - | - | 0.658  (0.194-2.228) | 0.501 |  |  | 2.115 (1.104-4.049) | 0.024 | - | - |
| Urethral Foley catheter | 1.022  (0.677-1.542) | 0.917 | - | - | 0.991  (0.577-1.700) | 0.973 | - | - | 0.730  (0.216-2.475) | 0.614 |  |  | 1.085 (0.496-2.373) | 0.838 | - | - |
| Cystostomy | 2.242  (1.324-3.799) | 0.003 | - | - | 0.974  (0.544-1.743) | 0.928 | - | - | 0.894  (0.263-3.308) | 0.858 |  |  | 1.340 (0.610-2.940) | 0.056 |  |  |

**Abbreviations:** VUDS= video urodynamic studies; AD= autonomic dysreflexia; DSD= detrusor sphincter dyssynergia; VUR= vesicourethral reflux; Pdet=detrusor pressure; DO= detrusor overactivity; DO-DU= detrusor overactivity with detrusor underactivity; DU/DA=detrusor underactivity/acontractile detrusor; CIC= clean intermittent catheterization; CKD/ESRD= chronic kidney disease/end stage renal disease.
